# Plant Oxidosqualene Metabolism: Cycloartenol Synthase–Dependent Sterol Biosynthesis in *Nicotiana benthamiana*

**DOI:** 10.1371/journal.pone.0109156

**Published:** 2014-10-24

**Authors:** Elisabet Gas-Pascual, Anne Berna, Thomas J. Bach, Hubert Schaller

**Affiliations:** Institut de Biologie Moléculaire des Plantes du CNRS & Université de Strasbourg, Institut de Botanique, Strasbourg, France; Simon Fraser University, Canada

## Abstract

The plant sterol pathway exhibits a major biosynthetic difference as compared with that of metazoans. The committed sterol precursor is the pentacyclic cycloartenol (9β,19-cyclolanost-24-en-3β-ol) and not lanosterol (lanosta-8,24-dien-3β-ol), as it was shown in the late sixties. However, plant genome mining over the last years revealed the general presence of lanosterol synthases encoding sequences (*LAS1*) in the oxidosqualene cyclase repertoire, in addition to cycloartenol synthases (*CAS1*) and to non-steroidal triterpene synthases that contribute to the metabolic diversity of C_30_H_50_O compounds on earth. Furthermore, plant LAS1 proteins have been unambiguously identified by peptidic signatures and by their capacity to complement the yeast lanosterol synthase deficiency. A dual pathway for the synthesis of sterols through lanosterol and cycloartenol was reported in the model *Arabidopsis thaliana*, though the contribution of a lanosterol pathway to the production of 24-alkyl-Δ^5^-sterols was quite marginal (Ohyama et al. (2009) *PNAS* 106, 725). To investigate further the physiological relevance of *CAS1* and *LAS1* genes in plants, we have silenced their expression in *Nicotiana benthamiana*. We used virus induced gene silencing (VIGS) based on gene specific sequences from a *Nicotiana tabacum CAS1* or derived from the solgenomics initiative (http://solgenomics.net/) to challenge the respective roles of *CAS1* and *LAS1*. In this report, we show a CAS1-specific functional sterol pathway in engineered yeast, and a strict dependence on CAS1 of tobacco sterol biosynthesis.

## Introduction

**Figure 1:**
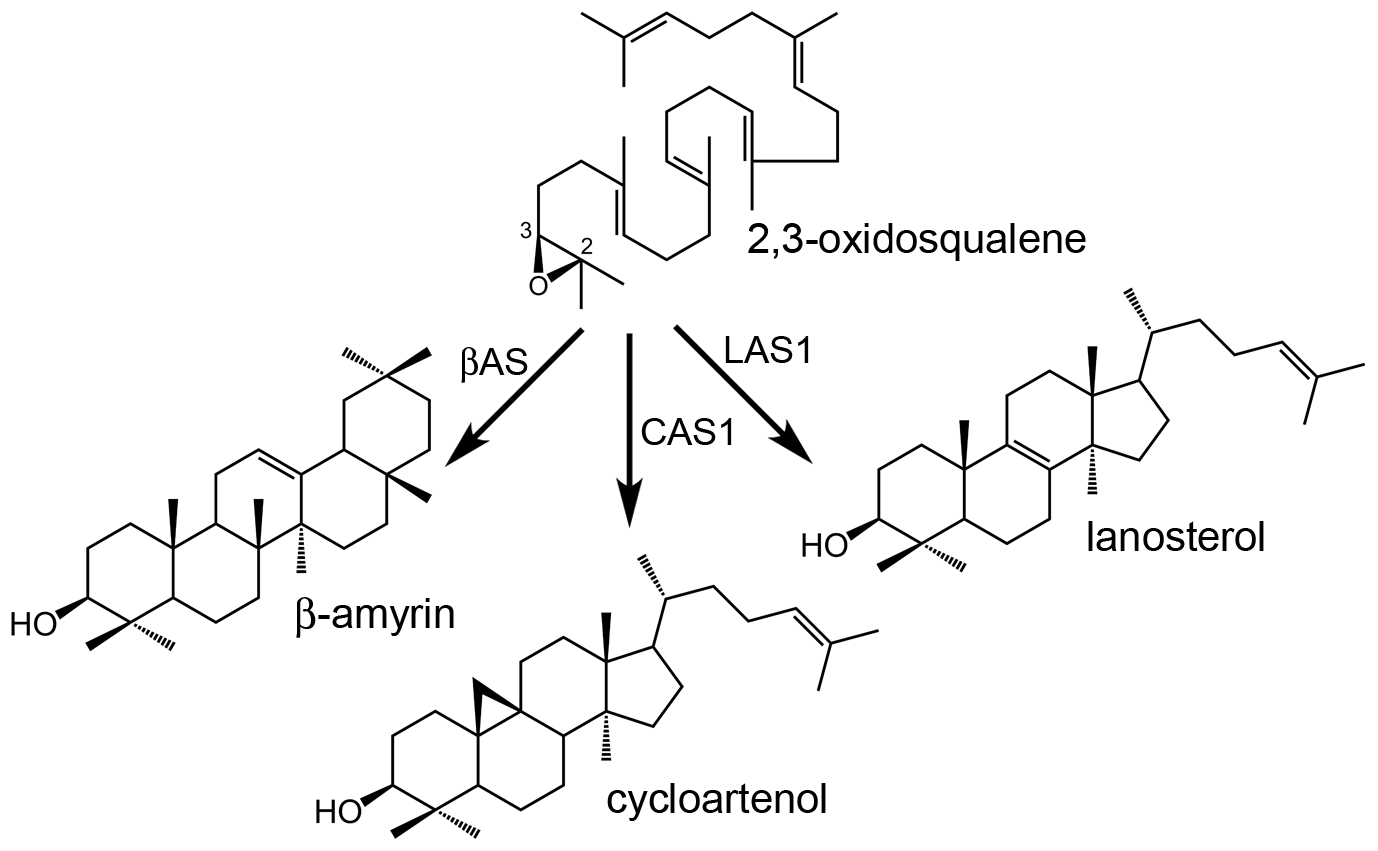
Prominent 2,3-oxidosqualene cyclization products. **1**, β-amyrin (olean-12-en-3β-ol); **2**, cycloartenol (9β,19-cyclolanost-24-en-3β-ol); **3**, lanosterol (lanosta-8,24-dien-3β-ol). βAMS, β-amyrin synthase; CAS1, cycloartenol synthase; LAS1, lanosterol synthase.

**Figure 2:**
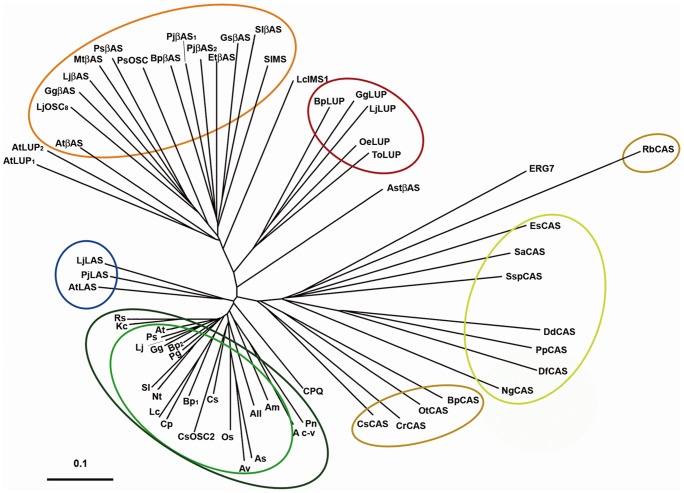
Phylogenetic tree illustrating the distribution of 2,3-oxidosqualene cyclases in plants, algae, and protists. The distance between each sequence was calculated using the program CLUSTAL W. The phylogenetic tree was drawn using Phylodendron online tool (http://iubio.bio.indiana.edu/treeapp/treeprint-form.html). Scale represents 0.1 amino acid substitutions per site. Protein clusters are enhanced by colors as follows: red and orange, β-amyrin and promiscuous β-amyrin synthases, dark red, lupeol synthases; blue, plant lanosterol synthases; green, plant cycloartenol synthases; brown, cycloartenol synthases from algae or a planctomycete, and yellow, cycloartenol synthases from protists. Abbreviations and accession numbers are given in Table S1. All proteins displayed in the phylogenetic tree have either been functionally characterized or genome annotated (except CAS1 from *Salpingoeca sp*, *Allium macrosternom*, *Avena strigosa*, *Avena ventricosa* and *Luffa cylindrica*, which have been annotated based on sequence homology and/or expression evidence).

Plants, algae and some protists synthesize their sterols through a biosynthetic route that contains a pentacyclic steroidal cyclization product of 2,3-oxidosqualene, namely, cycloartenol (9β,19-cyclolanost-24-en-3β-ol, Fig. 1) the product of the cycloartenol synthase (CAS1, EC 5.4.99.8) [1]–[5]. In contrast, other organisms like mammals and fungi use lanosterol (lanosta-8,24-dien-3β-ol, Fig. 1), an isomeric tetracyclic steroidal cyclization product of 2,3-oxidosqualene that is made by the lanosterol synthase (LAS1, EC 5.4.99.7). The post-squalene biosynthetic pathways leading to Δ^5^-sterols from cycloartenol or from lanosterol have been described comprehensively with respect to the enzymes implicated and to the chemistry of the reactions considered [6]–[8]. However, the rationale of the conserved biosynthetic detour through 9β,19-cyclopropyl sterol intermediates in plants, algae and protists is not understood [9]. Higher plants have in addition the capacity to produce a huge array of mostly dispensable non steroidal 2,3-oxidosqualene cyclization products (for instance β-amyrin, Fig. 1) generated by various triterpene synthases that have been classified into multiple families [10], [11]. The distribution of a representative set of oxidosqualene cyclases (OSCs) in a phylogenetic tree gives a clear view of main groups (Fig. 2, Table S1). The nearly ubiquitous presence of β-amyrin synthases and lupeol synthases that form one large group indicates that some triterpenes of the oleanane or lupane series may have a general physiological significance beyond the species-specific well-known accumulation of triterpene derivatives, such as saponins in *Gypsophila trichotoma* [12]. This has been described recently in a series of studies describing the effect of triterpene lipids on the structure of cuticular lipophilic barriers in *Arabidopsis thaliana* [13], or the role of these triterpenes as epicuticular crystals in the mediation of plant-insect interactions in a species-specific manner [14]. In addition, functional approaches *in planta* have assigned specific roles for some of these triterpene synthases in the production of β-amyrin as a precursor of phytoanticipins like the avenacin saponins in oat [15]. CAS1 proteins cluster into distinct entities indicating their appartenance to land plants, algae, or protists (Fig. 2). A distinct group of proteins is formed by OSCs that have been characterized as LAS1 from plants owing to the complementation of the yeast lanosterol synthase deficient *erg7* mutant [16]. The biochemical characterization of a wealth of triterpene synthases (or OSCs) isolated from many different plant species revealed in addition to CAS1 and OSCs that produce triterpenoids of diverse structure, the general presence of LAS1 in the plant OSCs repertoire [17], [18]. The increasing number of genome sequences available in the databases is constantly reinforcing this fact. The contribution of a lanosterol pathway to the production of 24-alkyl-Δ^5^-sterols was shown to be marginal even upon strong expression of *LAS1* in transgenic *Arabidopsis thaliana* [19]. Moreover, the contribution of lanosterol as a precursor of natural products remains unclear until now [20]. In fact, lanosterol has been only detected in large amounts in the latex of *Euphorbia* species [21]. The restriction of this sterol precursor to the complex *Euphorbia* genus has not yet received any functional explanation. It is nonetheless worth noting that 2-tritio lanosterol and 2-tritio cycloartenol when fed to *Sorghum bicolor* leaves were converted to cholesterol and sitosterol eventhough cycloartenol was a more effective sterol precursor than lanosterol [22]. Thus, the presence of a lanosterol metabolism in plants, possibly related to phytosterol biosynthesis, deserves further investigations.

The essential role of CAS1 in sterol biosynthesis and plant development was demonstrated by a series of *Arabidopsis thaliana* hypomorphic or conditional alleles [23]. Such plants developed an albino phenotype in tissues that undergo active cell division and elongation such as leaf margins and apices. The negative effect upon plastid development was strictly coincidental with an accumulation of 2,3-oxidosqualene. The CAS1-dependent sterol biosynthetic segment consists of a succession of 4 enzymatic steps leading from cycloartenol to cycloeucalenol (Fig. S1). This upstream segment is connected with the downstream segment leading to 24-alkyl-Δ^5^-sterols by the enzyme CPI (cyclopropyl isomerase, EC 5.5.1.9), being able to isomerize cycloeucalenol into the tetracyclic 4α-methyl sterol, obtusifoliol [24], [25]. Obtusifoliol is further converted into phytosterols in a way that is reminiscent of the conversion of lanosterol to ergosterol in metazoans [8], [9]. This CAS1 segment is highly conserved in photosynthetic organisms and beyond, in protists. The strong phenotype of an *Arabidopsis thaliana cpi1-1* mutant indicates that a normal life span cannot be attained with the CAS1 biosynthetic segment only [26], although growth of cells [27] and to a limited extent of plants with 9β,19-cyclopropylsterols [28] have been reported.

The co-existence of functional CAS1 and LAS1 in plants requires further studies. So far, *in planta* characterization of CAS1 and LAS1 was restricted to *Arabidopsis thaliana*. In order to further document dual cycloartenol and lanosterol pathways in higher plants we have isolated a *CAS1* from *Nicotiana tabacum* and studied its function in yeast and in *Nicotiana benthamiana*. We used virus induced gene silencing (VIGS) based on gene specific sequences derived from the solgenomics initiative (http://solgenomics.net/) to challenge the respective roles of *CAS1* and *LAS1* in *Nicotiana benthamiana*. In this report, we show a strict dependence on CAS1 of tobacco sterol biosynthesis.

## Results and Discussion

### Genes and cDNAs encoding 2,3-oxidosqualene cyclases (OSCs) in *Nicotiana tabacum* and *Nicotiana benthamiana*

We surveyed the genome databases to identify *Nicotiana* orthologs of CAS1 and LAS1. We then cloned a *Nicotiana tabacum CAS1* cDNA by recursive PCR using degenerated primers over conserved sequences. Primers used for that cloning procedure are given Table S2. The full length cDNA encoded a 2856 bp open reading frame displaying 78% identity with *AtCAS1* and consequently was named *NtCAS1* (GenBank accession KM452913). We next identified in the genome of *Nicotiana benthamiana* (http://solgenomics.net/) two genes whose products had 70% and 58% identity with AtCAS1 and AtLAS1, respectively. Relevant sequences identified in *Nicotiana* and in other Solanaceae are given in Table S3. It is worth noting that probing the genome of *Nicotiana benthamiana* with the functionally identified tomato triterpene synthases [29] resulted in the identification of a predicted protein (NbS00041716g0004.1) that exhibited 85% identity with the tomato β-amyrin synthase and 77% with the *Arabidopsis thaliana* β-amyrin synthase (Table S3). The presence of a putative β-amyrin synthase gene in the *Nicotiana benthamiana* genome certainly requires further investigations to determine its physiological relevance since β-amyrin has not been detected in *Nicotiana benthamiana* leaves in normal physiological conditions [30]. The simple structure of the OSC gene family in *Nicotiana benthamiana* makes this plant an excellent model to further investigate functional aspects related to *CAS1* and *LAS1* genes.

**Figure 3:**
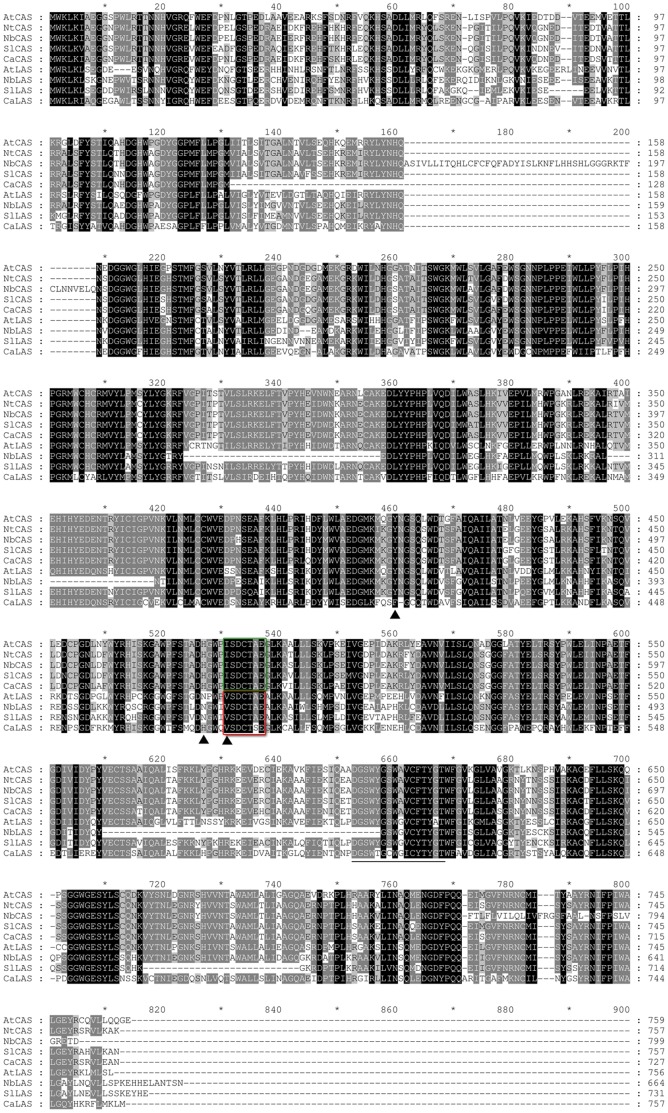
Alignment of selected 2,3-oxidosqualene-cycloartenol cyclases (CAS1) and 2,3-oxidosqualene-lanosterol cyclases (LAS1) from *solanaceae*. At, *Arabidopsis thaliana*; Nt, *Nicotiana tabacum*; Nb, *Nicotiana benthamiana*; Sl, *Solanum lycopersicon*; Ca, *Capsicum annuum*. Dashes are for gaps that maximize the alignment made with GeneDoc [48]. Conserved residues are highlighted in black or grey. The DCTAE motif is boxed (in green for CAS1; in red for LAS1). Important catalytic residues specifying cyclization of 2,3-oxidosqualene into cycloartenol or lanosterol are marked with arrowheads (Tyr 410, His 477 and Ile 481, *Arabidopsis thaliana* numbering). A terpene synthase signature DGSWyGsWAVcFtYG is underlined.

We cloned by RT-PCR a cDNA fragment from *N.benthamiana* leaf RNA, that corresponded to an expressed *NbLAS1* (Table S3), confirmed by qRT-PCR analysis. The alignement of amino acid sequences of CAS1 and LAS1 from *Nicotiana tabacum*, *N.benthamiana*, *Solanum lycopersicon* and *Capsicum annuum* (Fig. 3) shows features that support the identification of CAS1 and LAS1 sequences in these solanaceae. In fact, these two enzymes have been studied extensively. Molecular evolution and site-directed mutagenesis experiments revealed important aminoacid residues and conserved motifs that govern the cyclization of OSC into cycloartenol or into lanosterol. All CAS1 enzymes have a strict requirement for His477 and I481 (*Arabidopsis* numbering) in the vicinity of the conserved DCTAE motif implicated in substrate protonation, whereas all LAS1 proteins have a strict requirement for a V481 (Fig. 3) [31], [32]. A thorough analysis of the cyclization reactions, which required the heterologous expression of the plant CAS1 or LAS1 enzymes in the yeast *erg7* (lanosterol-deficient) mutant led to a current understanding of the catalytic differences in both reactions [33].

### Functional characterization of CAS1 and LAS1 in *Nicotiana benthamiana*

**Figure 4:**
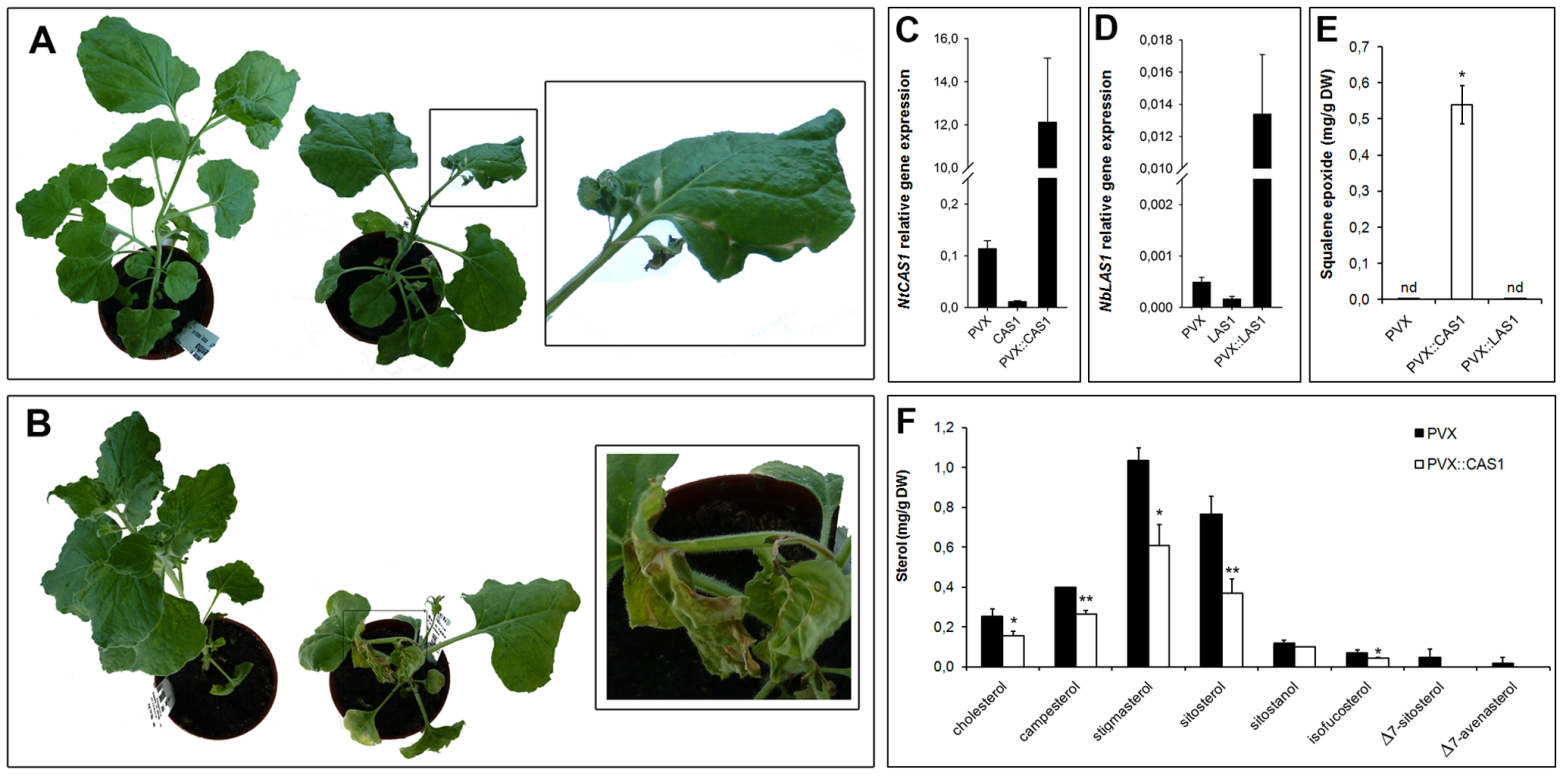
VIGS of CAS1 and LAS1 in *Nicotiana benthamiana*. **A**, Morphological phenotype of *PVX* (left) and *PVX::CAS1* (right) plants 4 weeks after inoculation, the close-up shows bleaching of veins. **B**, Morphological phenotype of *PVX* (left) and *PVX::CAS1* (right) plants 5 weeks after inoculation, the close-up shows leaf wilting and necrosis. **C**, Relative gene expression in *PVX::CAS1* plants, *CAS1* is a measurement of the endogenous *NbCAS1* level, *PVX::CAS1* is a measurement of the viral *NtCAS1* transcript. **D**, Relative gene expression in *PVX::LAS1* plants, *LAS1* is a measurement of the endogenous *NbLAS1* level, *PVX::LAS1* is a measurement of the viral *NbLAS1* transcript. **E**, squalene epoxide amounts measured by GC-FID in silenced plants. **F**, sterol composition of *PVX* and *PVX::CAS1* plants. Structure of the compounds detected here are shown in **Fig. S1**. The pictures in **A** and **B** are representative of 4 independent experiments that included all 3 plants inoculated with each type of viral transcripts.

To ascertain the function of CAS1 and LAS1 in *N. benthamiana*, we silenced the corresponding genes in expanding leaves of young plants using VIGS. For this we cloned gene-specific cDNA fragments of *NtCAS1* and *NbLAS1* into a PVX-based vector. Transcripts generated from *PVX*, *PVX::CAS1*, and *PVX::LAS1* constructs were then inoculated into leaves by wounding. Visual inspection of inoculated plants showed very clearly the appearance of a strong phenotype for *PVX::CAS1* plants. The first effects on growth were a general reduction of plant stature and a leaf bleaching that was prominent on veins (Fig. 4A). In the most severe cases, plant leaves and other tissues that had developed after the inoculation of the *PVX* transcripts to the first and second leaf pair displayed a strong wilting and necrotic zones (Fig. 4B). This impaired any further growth and development. Conversely, *PVX::LAS1* plants had a lifespan and morphological aspect identical to *PVX*- or mock-inoculated plants (Fig. S1). Gene expression analysis carried out by RT-qPCR indicated a massive accumulation of the viral transcripts *PVX::CAS1* and a reduction of the endogenous *CAS1* messenger RNA in *PVX::CAS1* plants (Fig. 4C). Closely similar results were obtained for the expression of *LAS1* that was reduced in *PVX::LAS1* plants concurrently to a huge load of viral *PVX::LAS1* transcripts (Fig. 4D). Next, measurements of squalene-derived products by gas chromatography revealed an accumulation of 2,3-oxidosqualene ranging from 0.5 up to 1 milligram per gram dry weight in *PVX::CAS1* leaf tissue, whereas the amount of that OSC substrate remained undetectable in *PVX::LAS1* plants, as it was the case for control *PVX* or mock plants (Fig. 4E). Total sterols decreased in *PVX::CAS1* plants, consistent with a blockage of a biosynthetic step upstream to cycloartenol (Fig. 4F). In contrast, the analysis by GC-MS of the sterol composition of *PVX::LAS1* plants did not show major changes when compared to control plants, indicating no major role of *LAS1* in sterol accumulation in *N. benthamiana*. In this study, quantification of both cycloartenol and lanosterol were attempted. Only trace amounts of cycloartenol were detected in some of the control samples analyzed in Fig. 4F and Fig. S2 (data not shown) but were often below the limit of detection. Finally, GC-MS analysis revealed in *PVX::CAS1* plants a very small amount of β-amyrin (Fig. S3). This is strongly in favour of the existence of a true β-amyrin synthase otherwise found as a gene sequence in the *N. benthamiana* genome (NbS00041716g0004.1, Table S3). The fact that 2,3-oxidosqualene strongly accumulated in *PVX::CAS1* leaf tissues most probably allowed this β-amyrin synthase to access to a pool of that substrate. It is worth noting that such a strong accumulation of 2,3-oxidosqualene precludes reliable enzymatic analyses using subcellular fractions.

### NtCAS1 defines a functional protist/plant sterol pathway in yeast

**Figure 5:**
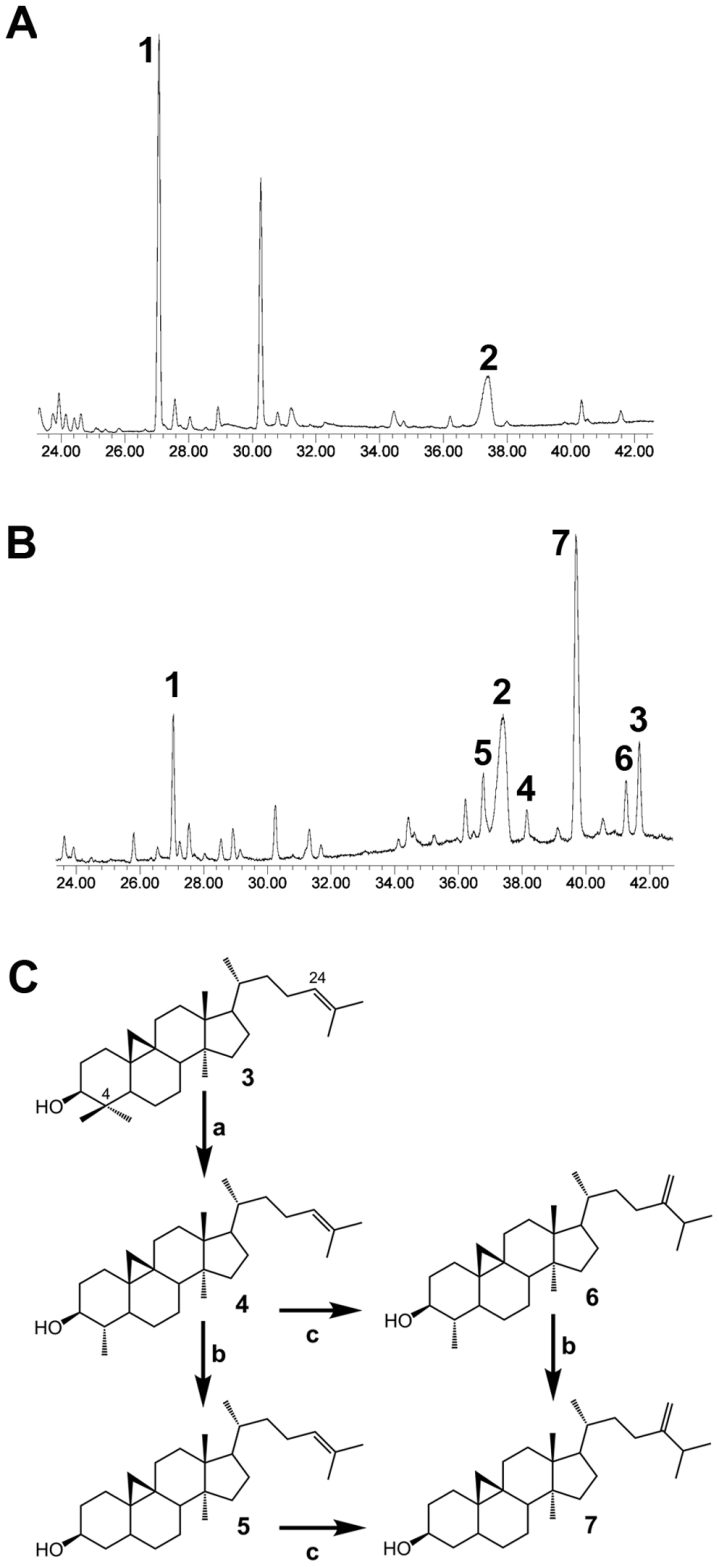
Sterol profile determind by GC-MS of *erg7* expressing a tobacco cycloartenol synthase CAS1. **A**, TIC of a total unsaponifiable extract of *erg7* transformed with a void vector. **B**, TIC of a total unsaponifiable extract of *erg7::NtCAS1*. **C**, 9β,19-cyclopropylsterol biosynthetic pathway in yeast. Compounds are: **1**, 2,3-oxidosqualene; **2**, ergosterol; **3**, cycloartenol; **4**, 31-norcycloartenol; **5**, 24-dehydropollinastanol; **6**, cycloeucalenol; **7**, 24-methylene pollinastanol. Compounds are identified according to their mass spectra and to those of authentic standards for 3, 6, and 7 purified from plant material as previously described [25], [26], [44]. Peaks that are not numbered are not sterols.

**Figure 6:**
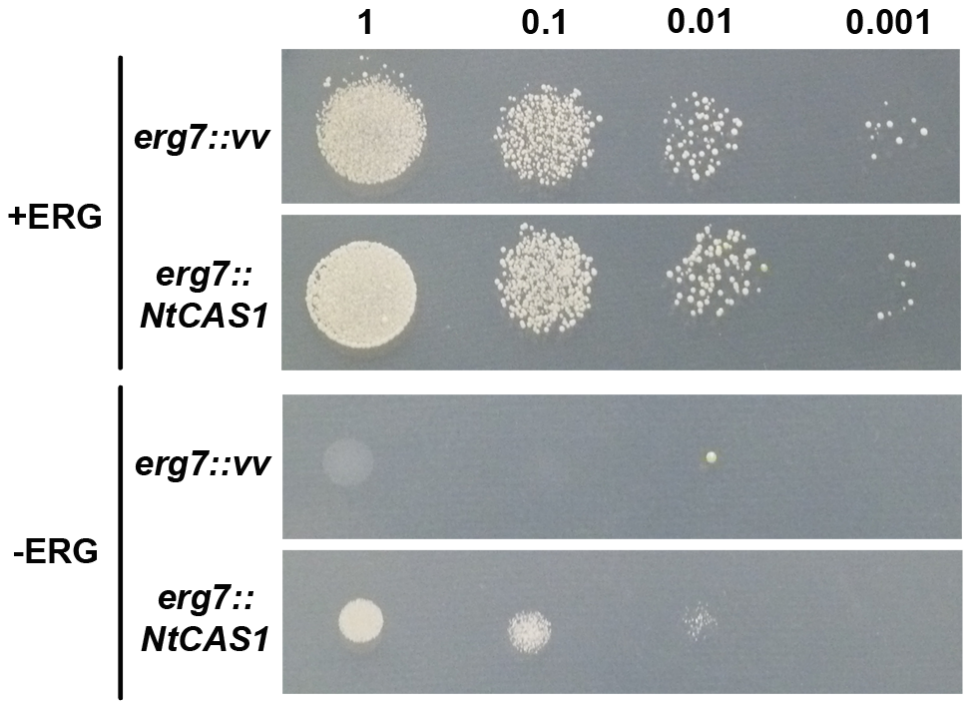
Yeast spotting assay with ERG7 deficient *gil77* or *gil77::CAS1* strains. Growth of *gil77* yeast strain transformed with the pYeDP60 vector or pYeDP60-*NtCAS1* was tested on SGal medium supplemented or not with 20 µg/ml of ergosterol. After 5 days at 30°C, plates supplemented with ergosterol showed fully-grown colonies, whereas inductive conditions (SGal without ergosterol) allowed only limited growth of yeast expressing NtCAS, indicating however that NtCAS1 expression partially overcomes ERG7 deficiency.

The functional identification of *NtCAS1* as encoding a cycloartenol synthase was further performed by its expression in the yeast mutant *erg7* that lacks an endogenous lanosterol synthase [34]. Upon galactose-induced expression of *NtCAS1*, yeast cells grown in the presence of exogenous ergosterol displayed a sterol profile (Fig. 5A and B) that included cycloartenol (peak 3) and four other sterols (peaks 4 to 7) in addition to ergosterol (peak 2). The major sterol was 24-methylene pollinastanol (peak 7) that accounted for 53% of the total and represented the most probable pathway end-product generated by the yeast steroidogenic machinery, apparently being versatile enough to accept cycloartenol as a substrate. The most probable sterol pathway of *erg7::NtCAS1* is shown in Fig. 5C. Cycloartenol is demethylated at C-4 by the demethylation complex [35] to produce 31-*nor*-cycloartenol, which is further taken by the sterol-C24-methyltransferase (ERG6) to produce cycloeucalenol (peak 4) or by the C-4 demethylation complex to remove the second methyl group at C-4 to yield 24-methylene pollinastanol (peak 7). The favored grid of possible interconversions presented in Fig. 5C is in agreement with previous work done with exogenously cycloartenol-fed yeasts [36] or more recently with the expression of a rice CAS1 in yeast, this to a certain extent [37]. The absence of 24-methylene cycloartanol (the direct product of a possible action of ERG6 on cycloartenol) may indicate that the recruitment of the yeast C4-demethylation enzyme complex (ERG25, ERG26, ERG27 and ERG28) occurs prior to the side chain alkylation, or that 24-methylene cycloartanol has a rapid turnover [38]. As it is the case for all enzymatic conversions implied in this pathway, the methylation at C24 was partial, which may explain the identification of 24-dehydropollinastanol as well (peak 6). Interestingly, no sterols bearing a reduced side chain at Δ^24^ were detected, indicating a probable exclusion of ERG4, the yeast Δ^24^-reductase, from the cellular compartment involved in the yeast 9β,19-cyclopropylsterol biosynthesis. In this respect, it was demonstrated over the last years that the yeast sterol biosynthetic segments are seemingly localized in distinct compartments, of which the lipid droplets are major players in hosting ERG6, ERG7 and the C-4 demethylation complex [39]. Thus, the sterol biosynthetic features of *erg7::NtCAS1* were identical to the 9β,19-cyclopropylsterol biosynthetic segment of protists, algae, or plants. Cell cultures of these latter biological models were shown to grow, albeit partially, only with that biosynthetic segment, as seen after chemical inhibition of the cyclopropyl isomerase that links the 9β,19-cyclopropylsterol biosynthetic segment to the downstream tetracyclic segment of the sterol pathway [27]. Indeed, those eukaryotic cells could live with 4,4-demethylated cycloartenol derivatives such as 24-methylene- and 24-methyl-pollinastanol. For these reasons, the growth of *erg7::NtCAS1* was examined on a minimal selective medium in the presence or absence of ergosterol (Fig. 6). Growth assessment performed as spotting assays was made after five days. In the presence of ergosterol, *erg7* or *erg7::NtCAS1* displayed a closely similar growth profile along a range of dilutions, whereas in the absence of ergosterol, only *erg7::NtCAS1* was able to grow significantly when compared to *erg7*. The growth was however slowed down as shown by the significantly reduced spots. This reinforces previous studies showing that cycloartenol cannot support growth of yeast unless it has been metabolized to C-4 desmethyl sterols such as 24,25-dehydropollinastanol [40]. Such result was in agreement as well with previous observations [27] about 9β,19-cyclopropylsterols being good surrogates to tetracyclic Δ^5^-sterols (cholesterol, ergosterol, sitosterol) in a way that they do not prevent a yeast cell or a plant cell to divide. Nonetheless, cellular and morphogenetic inhibitions of multicellular organisms depleted in 24-alkyl-Δ^5^-sterols were severe [23], [26].

## Conclusions

VIGS is a powerful tool to challenge the biological function of genes [41]. The most popular model plant that is currently the VIGS workhorse for biochemical studies is *Nicotiana benthamiana* (http://bti.cornell.edu/nicotiana-benthamiana/). Specific silencing of genes [42], analysis of plant-pathogen interactions mediated by sphingolipid biosynthesis [43], and genetic screens [44] have been described. We show here using VIGS that the tobacco cycloartenol synthase gene *CAS1* encodes the only OSC essential to sterol biosynthesis. Its decreased expression results in impaired development, bleaching of leaf veins, severe leaf wilting, vegetative and reproductive development arrest. This is in full agreement with previous studies that demonstrated the vital role of CAS1 in *Arabidopsis thaliana* owing to a genetic mosaic analysis [23]. Furthermore, the morphogenetic inhibition developed by *PVX::CAS1 Nicotiana benthamiana* leaves is closely similar to the severe stunting of *PVX::CYP51* plants, due to a lack of 24-alkyl-Δ^5^-sterols upon inhibition of *NtCYP51* that encodes obtusifoliol-14-demethylase [42]. However, the strong albinism that characterized leaf veins at the onset of silencing remained typical of *PVX::CAS1*, indicating a strict requirement of cycloartenol derivatives for proper development. Interestingly, a CAS1-dependent sterol biosynthesis supported yeast growth to a limited extent, that may indicate common features of plant and yeast cellular functions exerted by 9β,19-cyclopropylsterols. Finally, this work points out the mostly dispensable nature of LAS1 in *Nicotiana benthamiana*.

## Materials and Methods

### RT-PCR cloning of cDNAs

Total RNAs from 7-day-old TBY-2 cells or from *Nicotiana benthamiana* young leaves were extracted and purified with Trizol reagent (Invitrogen) or with the NucleoSpin RNA Plant kit (Macherey-Nagel), following the manufacturer's recommendations. *NtCAS1* full length cDNA obtained by recursive PCR cloning (primers in Table S2) was subcloned in pBluescript vectors and sequenced according to standard molecular biology methods.

### Virus-Induced Gene Silencing

Potato Virus X vectors were engineered to achieve the silencing of *Nicotiana benthamiana CAS1* and *LAS1* genes. A 1 kb fragment of the *NtCAS1* cDNA (nucleotides 958 to 2023 of the CDS, GenBank accession KM452914) was obtained by EcoRV digestion of the pBluescript*::NtCAS1* cloning vector. A 0.45 kb fragment designed from the first 470 nucleotides of the *NbLAS1* cDNA retrieved from the solgenomics database (http://solgenomics.net/, NbS00053226g0006) was isolated by RT-PCR using primers 5′-GGGAAACGAACCGTGGG-3′ (forward) and 5′-GGTGAACACAGTGTTATCA-3′ (reverse) formatted directionally into ClaI and SalI, respectively (GenBank accession KM452915). Purification and subcloning of cDNA fragments was done according to standard cloning procedures. The 1 kb *CAS1* fragment was inserted into the EcoRV site of the pP2C2S vector [45], the 0.45 kb *LAS1* fragment was subcloned into the ClaI (5′ end) and SalI (3′ end) sites of the same vector. To generate high amounts of PVX-derived transcripts, pP2C2S constructs (10 µg) were linearized via SpeI restriction and used as template for an in vitro transcription reaction that was done with the Ribomax large scale RNA production system T7 kit (Promega). Transcript formation was followed by UV spectrophotometry. *N. benthamiana* plants that had two pairs of true leaves were inoculated with a PVX-derived transcript preparation made of 5 µg of RNAs in 50 mM phosphate buffer containing 0.05% macaloid and 10 µg of yeast total RNA, in a final volume of 250 µL. Formulated RNAs were dropped over four leaves of a plant previously covered with little amounts of abrasive celite powder. Gentle scratching of leaves favored the infection of leaf tissues with PVX-derived RNAs.

### qPCR measurements

Total RNAs were extracted from leaf material using Trizol reagent (Invitrogen), following the manufacturer's recommendations. Template cDNAs for gene expression measurements were prepared by a RT reaction carried out on 1 µg of total RNAs and 200 ng of random hexamers in a final volume of 20 µL. Real-time qPCR reactions were run in a final volume of 20 µL containing gene specific primes (5 µM), 1 µL of a 20-fold dilution of a RT reaction, and 2× SYBRGreen mix (Eurogenetech). Amplification reactions were monitored with the GeneAmp5700 instrument and the SDS detection software (Applied Biosystems). Primers for *PVX::NtCAS1*, endogenous *NbCAS1* (http://solgenomics.net/, NbS00021029g0013), *PVX ::NbLAS1*, endogenous *NbLAS1*, and *NtACTIN* as a reference gene are given in Table S2. Primers were designed with Primer3 publicly available software then validated according to standard qPCR requirements (standard curves for PCR efficiency, amplification dissociation curves for specificity).

### Yeast transformation

*NtCAS1* cDNA was subcloned into the pYeDP60 yeast expression vector via EcoRI/BamHI restriction sites. Lanosterol synthase (ERG7) deficient *gil77* yeast strain [34] was transformed either with the empty vector or with the *NtCAS1* construct. *Saccharomyces cerevisiae* transformations were performed using the lithium acetate procedure as described previously [46]. Transformed *erg7* yeast strains were selected for uracil prototrophy and grown at 30°C in the presence of exogenous ergosterol (5α-ergosta-5,7,22-trien-3β-ol, Sigma, 20 µg/mL). Expression of the inducible gene construct was turned on by culturing transformed yeast into a minimal medium supplemented with ergosterol and galactose (2%) for 1 day.

### Yeast spotting assays

Isolated colonies of either pYeDP60 void vector or pYeDP60-NtCAS1 were grown overnight in minimal liquid medium (SGI supplemented with ergosterol). Cells were harvested, washed and resuspended in sterile water to reach an OD_600_ = 1. Serial dilutions as 1∶10, 1∶100, and 1∶1000 were prepared in water and 5 µL each were spotted onto inductive minimal medium (SGal) plates either supplemented or not with exogenous ergosterol (20 µg/ml), and grown at 30°C for 5 days.

### Sterol and triterpene extraction and analysis

Freeze-dried yeast or plant samples (50 to 100 mg) were saponified for one hour in a 6% potassium hydroxyde methanolic solution at 80°C. The unsaponifiable fraction was extracted with hexane then filtrated through a 0.45 µm PFTE membrane (Pall Life Sciences). The dried extract was acetylated in toluene with a mixture of pyridine/acetic anhydride. After evaporation of the reagents, a known amount of betuline diacetate was added as an internal standard for GC-based quantification. Compound quantification included a t-test calculation. GC-FID used for quantification was performed with a 3400CX gas chromatograph (Varian) equipped with a DB-5 column (30 m wall-coated open tubular, 0.32 mm i.d., 250 µm film thickness, H_2_ flow rate 2 mL/min). The temperature of the injector and detector was 250°C and 300°C, respectively. Compounds were identified by GC-MS using a 5973N instrument (Agilent) equipped with a DB5-MS column and coupled to a 6853 mass analyser (Agilent). The temperature program of ovens included a steep ramp at 30°C/min from 60°C to 220°C then a 2°C/min increase from 220°C to 300°C. Sterols were unequivocally identified by coincidental retention time and identical EI-MS spectra at 70 eV like reference compounds as described [47].

## Supporting Information

Figure S1 **Plant sterol biosynthetic pathway.** The biosynthetic segment framed in red is the so-called 9β,19-cyclopropylsterol segment that requires four enzymes from cycloartenol : SMT1, cycloartenol-C24-methyltransferase [S1]; SMO1, sterol methyl oxidase 1 [S2]; 3βHSD/D, 3β-hydroxysteroid/C4-decarboxylase [S3]; SR, sterone reductase [S4]. The CPI1, cyclopropyl isomerase, links the 9β,19-cyclopropylsterol biosynthetic segment to the metazoan-type sterol pathway that leads to 24-alkyl-Δ^5^-sterols (campesterol and sitosterol). Uncomplete methylation of the sterol side chain by SMT1 leads to the cholesterol pathway (not shown here for clarity of the figure).
(TIF)

Figure S2 **Morphological phenotype of (A)** ***PVX::LAS1*** **and (B)** ***PVX::CAS1*** **plants 4 weeks after inoculation.** The picture is representative of 4 independent experiments that included all 3 plants inoculated with each type of viral transcripts. The distribution of sterols in control *PVX*, *PVX::CAS1* or *PVX::LAS1* is shown in (C).
(TIFF)

Figure S3 **GC-MS analysis of** ***PVX*** **,** ***PVX ::LAS1*** **, and** ***PVX ::CAS1*** **unsaponifiable acetylated extracts showing the presence of β-amyrin only in** ***PVX ::CAS1*** **.** TIC between 22 min and 39 min are shown for (A), *PVX*; (B), *PVX ::LAS1*, (C), *PVX ::CAS1*. Compounds (as acetate derivatives) are : 1, cholesterol; 2, campesterol (the shouldering peak is 24-methylene cholesterol); 3, stigmasterol; 4, sitosterol; 5, isofucosterol; 6, 2,3-oxidosqualene; 7, β-amyrinat RT = 37,8 min. The mass spectrum of peak 7 and of authentic β-amyrin is shown in (D). β-amyrin has a mass spectrum with a typical ratio of m/z = 203 to m/z = 218 as discussed in supplemental reference [S5].
(PDF)

Table S1 **Gene nomenclature and protein references used to generate** **Figure 2** **.**
(PDF)

Table S2 **Primers used for NtCAS1 cloning and for qPCR measurements of CAS1 and LAS1.**
(PDF)

Table S3 **The Solanaceae OSC signatures and gene references.**
(PDF)

References S1 (DOCX)
