# Supplementary figures and images for: Plant Oxidosqualene Metabolism: Cycloartenol Synthase–Dependent Sterol Biosynthesis in Nicotiana benthamiana
Source: PLoS One. 2014 Oct 24;9(10):e109156. doi: 10.1371/journal.pone.0109156 (PMC4208727; doi:10.1371/journal.pone.0109156)

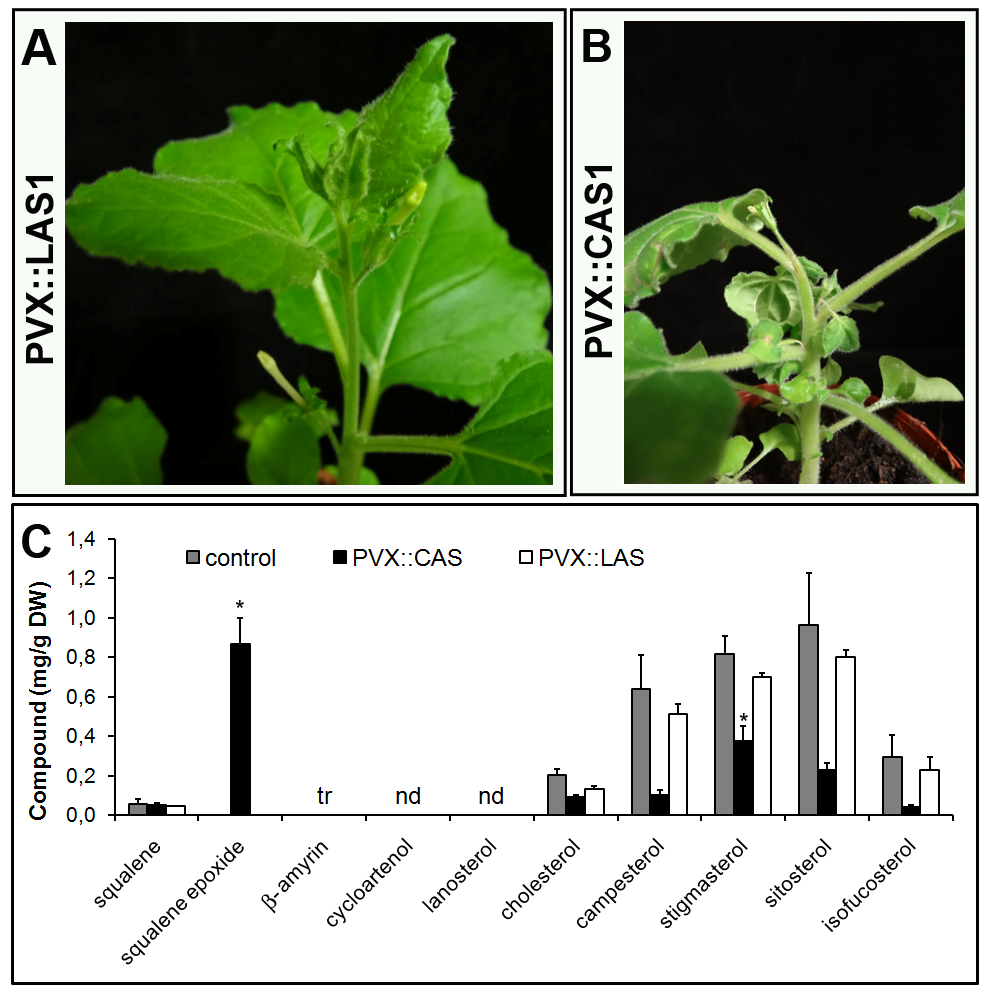

Supplement: Figure S2 — Morphological phenotype of (A) PVX::LAS1 and (B) PVX::CAS1 plants 4 weeks after inoculation. The picture is representative of 4 independent experiments that included all 3 plants inoculated with each type of viral transcripts. The distribution of sterols in control PVX, PVX::CAS1 or PVX::LAS1 is shown in (C). (TIFF) [file pone.0109156.s002.tiff]

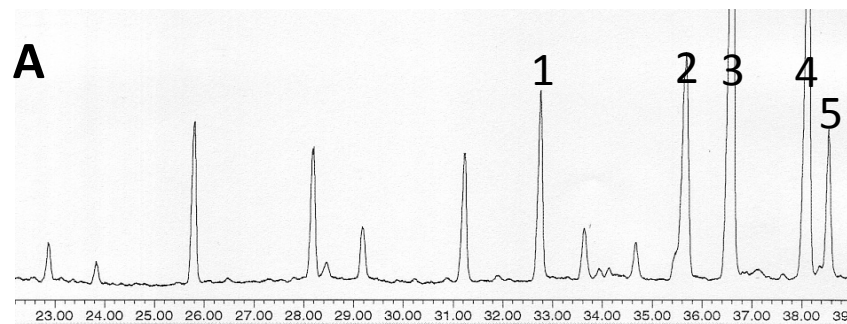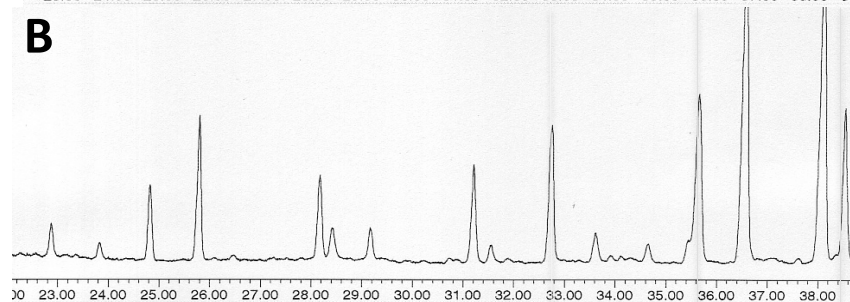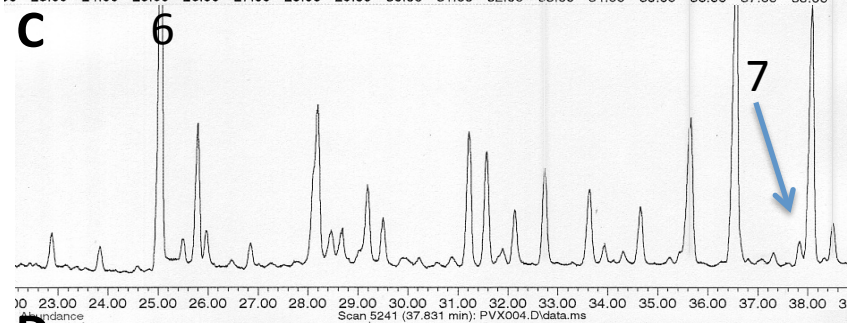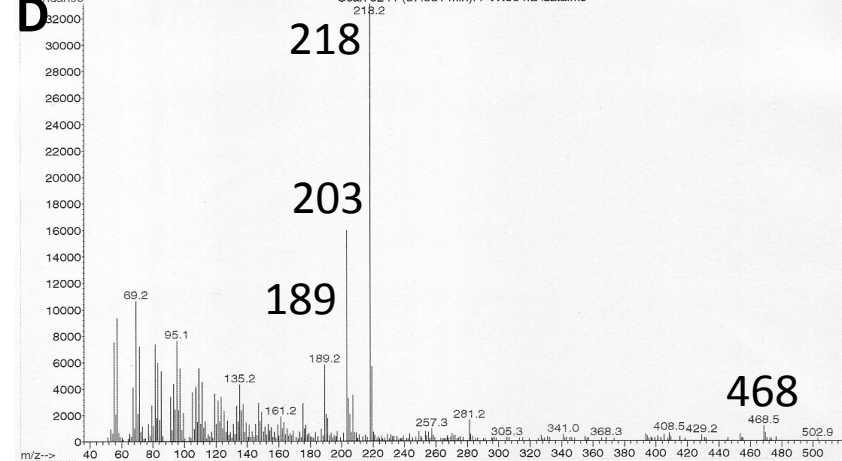

Supplement: Figure S3 — GC-MS analysis of PVX , PVX ::LAS1 , and PVX ::CAS1 unsaponifiable acetylated extracts showing the presence of β-amyrin only in PVX ::CAS1 . TIC between 22 min and 39 min are shown for (A), PVX; (B), PVX ::LAS1, (C), PVX ::CAS1. Compounds (as acetate derivatives) are : 1, cholesterol; 2, campesterol (the shouldering peak is 24-methylene cholesterol); 3, stigmasterol; 4, sitosterol; 5, isofucosterol; 6, 2,3-oxidosqualene; 7, β-amyrinat RT = 37,8 min. The mass spectrum of peak 7 and of authentic β-amyrin is shown in (D). β-amyrin has a mass spectrum with a typical ratio of m/z = 203 to m/z = 218 as discussed in supplemental reference [S5]. (PDF) [file pone.0109156.s003.pdf]
