# Supplementary material for: Plant Oxidosqualene Metabolism: Cycloartenol Synthase–Dependent Sterol Biosynthesis in Nicotiana benthamiana
Source: PLoS One. 2014 Oct 24;9(10):e109156. doi: 10.1371/journal.pone.0109156 (PMC4208727; doi:10.1371/journal.pone.0109156)
Supplement: Table S1 — Gene nomenclature and protein references used to generate Figure 2 . (PDF) [file pone.0109156.s004.pdf]

Table S1. Oxidosqualene cyclases used in Figure 2: Organism, Genbank accession number, OSC main products and references.

Fig 2 name	Complete name	Species	Group	GenBank #	Products (product identification method)	Other names	References
AtLUP1	mixed synthase	*Arabidopsis thaliana*	eudicots	AAD05032	Lupeol, 3b,20-dihydroxylupane, and minor products (GC-MS, NMR)	At1g78970/LUP1	Herrera [1]
AtLUP2	mixed synthase	*Arabidopsis thaliana*	eudicots	AAB94341	β-Amyrin, taraxasterol, and minor products (HPLC, NMR, GC-MS, LC-MS)	At1g78960/LUP2	Husselstein-Muller [2]; Kushiro [3]
AtβAS	β-amyrin synthase	*Arabidopsis thaliana*	eudicots	BAG82628	β-Amyrin (NMR, LC-MS)	At1g78950/AtBAS/LUP4	Shibuya [4]
LjOSC8	mixed synthase	*Lotus japonicus*	eudicots	AAO33580	Lupeol, β-amyrin and additional uncharacterized (HPLC)	AMY2/OSC8	Iturbe-Ormaetxe [5]
GgβAS	β-amyrin synthase	*Glycirrhiza glabra*	eudicots	BAA89815	β-Amyrin (LC-MS)	GgbAS1	Hayashi [6]
LjβAS	β-amyrin synthase	*Lotus japonicus*	eudicots	BAE53429	β-Amyrin (HPLC, EI-MS)	OSC1/AMY1	Sawai [7]
MtβAS	β-amyrin synthase	*Medicago truncatula*	eudicots	CAD23247	β-Amyrin (GC-MS, NMR, HPLC)	MtAMY1/bAS1	Suzuki [8]; Iturbe-Ormaetxe [5]
PsβAS	β-amyrin synthase	*Pisum sativum*	eudicots	BAA97558	β-Amyrin (LC-MS, GC-MS, NMR)	PSY	Morita [9]
PsOSC	mixed synthase	*Pisum sativum*	eudicots	BAA97559	α-Amyrin, β-amyrin, and minor products (LC-MS, GC-MS, NMR)	PSM	Morita [9]
BpβAS	β-amyrin synthase	*Betula platyphylla*	eudicots	BAB83088	β-Amyrin (LC-MS)	BPY	Zhang [10]
PgβAS1	β-amyrin synthase	*Panax ginseng*	eudicots	BAA33461	β-Amyrin (HPLC, MS, NMR)	PNY1	Kushiro [11,12]
PgβAS2	β-amyrin synthase	*Panax ginseng*	eudicots	BAA33722	β-Amyrin (HPLC, MS, NMR)	PNY2	Kushiro [12]
EtβAS	β-amyrin synthase	*Euphorbia tirucalli*	eudicots	BAE43642	β-Amyrin (GC-MS, NMR)	EtAS	Kajikawa [13]
SlβAS	β-amyrin synthase	*Solanum lycopersicum*	eudicots	ADU52574	β-amyrin synthase (GC-MS)	SlTTS1	Wang [14]
SlMS	mixed synthase	*Solanum lycopersicum*	eudicots	ADU52575	δ-amyrin synthase and 6 other products (GC-MS)	SlTTS2	Wang [14]
GsβAS	β-amyrin synthase	*Gentiana straminea*	eudicots	ACO24697	β-Amyrin (GC-MS)	BAS/AS1	Liu [15]
LcIMS1	Isomultiflorenol	*Luffa cylindrica*	eudicots	BAB68529	Isomultiflorenol (LC-MS)	LcIMS1	Hayashi [16]
BpLUP	lupeol synthase	*Betula platyphylla*	eudicots	BAB83087	Lupeol (LC-MS)	BPW	Zhang [10]
GgLUP	lupeol synthase	*Glycirrhiza glabra*	eudicots	BAD08587	Lupeol (LC-MS)	GgLUS1	Hayashi [17]
LjLUP	lupeol synthase	*Lotus japonicus*	eudicots	BAE53430	Lupeol (HPLC)	OSC3	Sawai [7]
OeLUP	lupeol synthase	*Olea europaea*	eudicots	BAA86930	Lupeol (LC-MS, NMR)	OEW	Shibuya [18]
ToLUP	lupeol synthase	*Taraxacum officinale*	eudicots	BAA86932	Lupeol (LC-MS, NMR)	TRW	Shibuya [18]
AstβAS	β-amyrin synthase	*Avena strigosa*	monocots	CAC84558	β-Amyrin (Genetic analysis, HPLC)	AsbAS1	Haralampidis [19]
ERG7	lanosterol synthase	*Saccharomyces cerevisiae*	fungi	AAA16975	Lanosterol (in vitro reaction, HPLC)	ERG7	Corey [20]
RbCAS	cycloartenol synthase	*Rhodopirellula baltica* SH 1	planctomycete	NP_869736	putative cycloartenol synthase based on whole genome sequence	predicted	Glöckner [21]
EsCAS	cycloartenol synthase	*Ectocarpus siliculosu*	brown algae	CBN75619	putative cycloartenol synthase based on whole genome sequence	predicted	Cock [22]
SaCAS	cycloartenol synthase	*Stigmatella aurantiaca DW4/3-1*	amoebozoa	CAD39196	Cycloartenol (GC-MS, NMR)	Stigmatella CAS	Bode [23]
SspCAS	cycloartenol synthase	*Salpingoeca sp. ATCC 50818*	amoebozoa	EGD81137	putative cycloartenol synthase based on sequence homology	predicted	[24]
DdCAS	cycloartenol synthase	*Dictyostelium discoideum*	amoebozoa	AAF80384	Cycloartenol (GC-MS, NMR)		Godzina [25]
PpCAS	cycloartenol synthase	*Polysphondylium pallidum PN500*	amoebozoa	EFA81207	putative cycloartenol synthase based on whole genome sequence	predicted	Heidel [26]
DfCAS	cycloartenol synthase	*Dictyostelium fasciculatum*	amoebozoa	EGG18936	putative cycloartenol synthase based on whole genome sequence	predicted	Heidel [26]

Table S1 (cont).

Fig 2 name	Complete name	Species	Group	GenBank #	Products (product identification method)	Other names	References
NgCAS	cycloartenol synthase	*Naegleria gruber*	amoebozoa	EFC50118	putative cycloartenol synthase based on whole genome sequence	predicted	Fritz-Laylin [27]
BpCAS	cycloartenol synthase	*Bathycoccus prasino*	picoalgae	CCO17148	putative cycloartenol synthase based on whole genome sequence	predicted	Vaulot [28]
OtCAS	cycloartenol synthase	*Ostreococcus tauri*	algae	CAL52737	putative cycloartenol synthase based on whole genome sequence	predicted	Derelle [29]
CrCAS	cycloartenol synthase	*Chlamydomonas reindhartii*	algae	EDP09612	putative cycloartenol synthase based on whole genome sequence	predicted	Merchant [30]
CsCAS	cycloartenol synthase	*Coccomyxa subellipsoidea* C-169	algae	EIE20900	putative cycloartenol synthase based on whole genome sequence	predicted	Blanc [31]
CPQ	cucurbitadienol synthase	*Cucurbita pepo*	eudicots	BAD34645	Cucurbitadienol (LC-MS, NMR)	CPQ	Shibuya [32]
Pn	cycloartenol synthase	*Polypodiodes niponica*	ferns	BAI48072	Cycloartenol (TLC, NMR)	PnCAS	Shinozaki [33]
A c-v	cycloartenol synthase	*Adiantus capitis-veneris*	ferns	BAF93208	Cycloartenol (LC-MS)	ACX	Shinozaki [34]
Am	cycloartenol synthase	*Abies magnifica*	gymnosperms	AAG44096	Cycloartenol (NMR, GC)	CAS1	Herrera [35]
All	cycloartenol synthase	*Allium macrosternom*	monocots	BAA84603	putative cycloartenol synthase based on sequence homology	Ama OSC	You [36]
As	cycloartenol synthase	*Avena strigosa*	monocots	CAC84559	putative cycloartenol synthase based on sequence homology	AsCS1	Haralampidis [19] ; Qi [37]
Av	cycloartenol synthase	*Avena ventricosa*	monocots	AAT38892	putative cycloartenol synthase based on sequence homology		Qi [37]
Os	cycloartenol synthase	*Oryza sativa*	monocots	BAH00370	Cycloartenol (GC-MS, NMR)	OsOSC1	Ito [38]
Cs	cycloartenol synthase	*Costus speciosus*	eudicots	BAB83253	Cycloartenol (LC-MS)	CSOSC1/CSI	Kawano [39]
CsOSC2	mixed synthase	*Costus speciosus*	eudicots	BAB83254	Lupeol, germanicol, β-amyrin and additional uncharacterized (LC-MS)	CSOSC2/CSV	Kawano [39]
Bp1	cycloartenol synthase	*Betula platyphylla*	eudicots	BAB83085	Cycloartenol (LC-MS)	BPX1	Zhang [10]
Cp	cycloartenol synthase	*Cucurbita pepo*	eudicots	BAD34644	Cycloartenol (LC-MS)	CPX	Shibuya [32]
Lc	cycloartenol synthase	*Luffa cylindrica*	eudicots	BAA85266	putative cycloartenol synthase based on sequence homology	LcCAS1	Hayashi [40]
Nt	cycloartenol synthase	*Nicotiana tabacum*	eudicots	for submission	yeast complemention (GC-MS)	NtCAS1	This work
Sl	cycloartenol synthase	*Solanum lycopersicum*	eudicots	NP_001233784	putative cycloartenol synthase based on whole genome sequence	Solyc04g070980	The tomato genome consortium [41]
Pg	cycloartenol synthase	*Panax ginseng*	eudicots	BAA33460	Cycloartenol (HPLC, *in vitro* activity)	PNX	Kushiro [11]
Bp2	cycloartenol synthase	*Betula platyphylla*	eudicots	BAB83086	Cycloartenol (LC-MS)	BPX2	Zhang [10]
Gg	cycloartenol synthase	*Glycirrhiza glabra*	eudicots	BAA76902	Cycloartenol (yeast complementation)	GgCAS1	Hayashi [42]
Lj	cycloartenol synthase	*Lotus japonicus*	eudicots	BAE53431	Cycloartenol (HPLC-EI-MS)	OSC5	Sawai [7]
Ps	cycloartenol synthase	*Pisum sativum*	eudicots	BAA23533	Cycloartenol (yeast complementation)	CASPEA	Morita [43]
At	cycloartenol synthase	*Arabidopsis thaliana*	eudicots	AAC04931	Cycloartenol (NMR, IR, MS)	At2g07050/CAS1	Corey [44]
Kc	cycloartenol synthase	*Kandelia candel*	eudicots	BAF73930	Cycloartenol (GC-MS)	Kca CAS1	Basyuni [45]
Rs	cycloartenol synthase	*Rhizophora stylosa*	eudicots	BAF73929	Cycloartenol (GC-MS)	Rst CAS1	Basyuni [45]
AtLAS	lanosterol synthase	*Arabidopsis thaliana*	eudicots	BAE95408	Lanosterol (GC-MS, NMR)	At3g45130/LSS1/PEN7	Kolesnikova [46], Suzuki [47]
PjLAS	lanosterol synthase	*Panax ginseng*	eudicots	BAA33462	Lanosterol (yeast complementation)	OSCPNZ1	Suzuki [47]
LjLAS	lanosterol synthase	*Lotus japonicus*	eudicots	BAE95410	Lanosterol (yeast complementation)	OSC7	Sawai [48]
