# Supplementary material for: Plant Oxidosqualene Metabolism: Cycloartenol Synthase–Dependent Sterol Biosynthesis in Nicotiana benthamiana
Source: PLoS One. 2014 Oct 24;9(10):e109156. doi: 10.1371/journal.pone.0109156 (PMC4208727; doi:10.1371/journal.pone.0109156)
Supplement: Table S2 — Primers used for NtCAS1 cloning and for qPCR measurements of CAS1 and LAS1. (PDF) [file pone.0109156.s005.pdf]

Table S2. Primers used for NtCAS1 cloning and for qPCR measurements of CAS1 and LAS1

Primers used for NtCAS cloning

**Name**	**Sequence**
NtCAS1 for	AGGAAGAYYTDTACTAYCCWCAYC
NtCAS1 rev	CGRTATTCTCCHARNGCCCA
CAS 5' for 1	GNAVTCCATGGCTHMGRWSNNY
CAS 5' for 2	ATGCGVMTDCAGTTYDCVAADGA
CAS 5' rev 1	GAATTCTTGGAAGGTGCAACT
CAS 5' rev 2	GTCATGAATTCTTGGAAGGTG
CAS 5’ RACE rev1	CTGGATCAAACTCCCATAGTTCCCTACC
CAS 5’ RACE rev2	TGCTCAAAGCGATGTTTGTGGAAATGCT
5′ RACE anchor primer	CUACUACUACUAGGCCACGCGTCGACTAGTAC$(GGGII)_3$G
Universal amplification primer	CUACUACUACUAGGCCACGCGTCGACTAGTAC
CAS 3' RACE for 2	CTTGAAGGCAACAGATATCATG
CAS 3' RACE for 3	CTGCAGGAAGGAACTACACTAA
3' RACE oligodT	GACTCGAGTCGACATCGATTTTTTTTTTTTTTTT
RACE 3’ primer	GCTGTCAACGATACGCTACGTAACG

Primers used for qPCR

**Name**	**Sequence**
NbCAS1 For	ATAGCAGAAGGAGGGAATCCA
NbCAS1 Rev	ACGATGTTTGTGGAAATGCTC
PVX::NtCAS1 For	CCTTCCAATTCATCCTGGAA
PVX::NtCAS1 Rev	GCCAACGAACCTTTTACCAT
NbLAS1 For	TGGATTCTTGAACATGGTGGT
NbLAS1 Rev	TTCGACCTGGGTGTATAGGAA
PVX::NbLAS1 For	TCGGTTTCATGTCAAACACAG
PVX::NbLAS1 Rev	TGCCCTTCTCAATGTTGTCTT
ACTIN For	TGATAACGGAACAGGAATGG
ACTIN Rev	TCGAGGTCGACCAACAATAC
