# Supplementary material for: Plant Oxidosqualene Metabolism: Cycloartenol Synthase–Dependent Sterol Biosynthesis in Nicotiana benthamiana
Source: PLoS One. 2014 Oct 24;9(10):e109156. doi: 10.1371/journal.pone.0109156 (PMC4208727; doi:10.1371/journal.pone.0109156)
Supplement: Table S3 — The Solanaceae OSC signatures and gene references. (PDF) [file pone.0109156.s006.pdf]

Species	%ID to *AtCAS1*	Cycloartenol synthase gene reference	DCTAE motif	%ID to *AtLAS1*	Lanosterol synthase gene reference	DCTAE motif	%ID to *AtBAS*	β-amyrin synthase gene reference	DCTAE motif
Arabidopsis thaliana	100	At2g07050	ISDCTAE	100	At3g45130	VSDCTAE	100	At1g78950	VSDCTAH
Nicotiana tabacum	78	This work	ISDCTAE	-			-		
Nicotiana benthamiana	70	NbS00021029g0013	ISDCTAE	58	NbS00053226g0006	VSDCTAE	77	NbS000417116g004	VSDCTAE
Solanum lycopersicon	75	Solyc04g070980	ISDCTAE	64	Solyc05g047580	VSDCTAE	83	Solyc12g006530	VSDCTAE
Capsicum annuum	75	Ca04g10740	ISDCTAE	55	Ca07g0900	VSDCTSE	73	Ca09g14750	VSDTTAE
