# Supplementary material for: Plant Oxidosqualene Metabolism: Cycloartenol Synthase–Dependent Sterol Biosynthesis in Nicotiana benthamiana
Source: PLoS One. 2014 Oct 24;9(10):e109156. doi: 10.1371/journal.pone.0109156 (PMC4208727; doi:10.1371/journal.pone.0109156)
Supplement: References S1 — (DOCX) [file pone.0109156.s007.docx]

**SI REFERENCES**

S1 Schaeffer A, Bouvier-Navé P, Benveniste P, Schaller H (2000) [Plant sterol-C24-methyl transferases: different profiles of tobacco transformed with SMT1 or SMT2.](http://www.ncbi.nlm.nih.gov/pubmed/10783003) Lipids 35 : 263-269.

# S2 Darnet S, Rahier A (2004) Plant sterol biosynthesis: identification of two distinct families of sterol 4alpha-methyl oxidases. Biochem J 378 : 889-898.

S3 Rahier A, Darnet S, Bouvier F, Camara B, Bard M (2006) [Molecular and enzymatic characterizations of novel bifunctional 3beta-hydroxysteroid dehydrogenases/C-4 decarboxylases from Arabidopsis thaliana.](http://www.ncbi.nlm.nih.gov/pubmed/16835224) J Biol Chem 281 : 27264-72727.

S4 Pascal S, Taton M, Rahier A (1994) [Plant sterol biosynthesis: identification of a NADPH dependent sterone reductase involved in sterol-4 demethylation.](http://www.ncbi.nlm.nih.gov/pubmed/8031136) Arch Biochem Biophys 312 : 260-271.

# S5 Elder JW, Benveniste P, Fonteneau P (1977) *In vitro* cyclization of squalene 2,3-epoxide to α-amyrin by microsomes from bramble cell suspension cultures. *Phytochemistry* 16 : 490-492.
